# Effects of a low-head weir on multi-scaled movement and behavior of three riverine fish species

**DOI:** 10.1038/s41598-020-63005-8

**Published:** 2020-04-22

**Authors:** Luke Carpenter-Bundhoo, Gavin L. Butler, Nick R. Bond, Stuart E. Bunn, Ivars V. Reinfelds, Mark J. Kennard

**Affiliations:** 1 Australian Rivers Institute, Griffith University, Nathan, Queensland 4111 Australia; 2 Department of Primary Industries NSW, Grafton Fisheries Centre, PMB 2, Grafton, NSW 2460 Australia; 3 Centre for Freshwater Ecosystems, La Trobe University, Wodonga, Victoria 3690 Australia; 4 NSW Office of Water, PO Box 53, Wollongong, NSW 2500 Australia

**Keywords:** Ecology, Behavioural ecology, Freshwater ecology

## Abstract

Despite providing considerable benefits to society, dams and weirs threaten riverine ecosystems by disrupting movement and migration of aquatic animals and degrading riverine habitats. Whilst the ecological impacts of large dams are well studied, the ecological effects of low-head weirs that are periodically drowned out by high flows are less well-understood. Here we examine the effects of a low-head weir on fine- and broad-scale movements, habitat use, and breeding behaviour of three species of native freshwater fish in the Nymboida River in coastal eastern Australia. Acoustic telemetry revealed that eastern freshwater cod (*Maccullochella ikei*) and eel-tailed catfish (*Tandanus tandanus*) made few large-scale movements, but Australian bass (*Percalates novemaculeata)* upstream of the weir were significantly more mobile than those below the weir. Within the weir pool, all three species displayed distinctive patterns in fine-scale movement behaviour that were likely related the deeper lentic environment created by the weir. No individuals of any species crossed the weir during the study period. *Tandanus tandanus* nesting behaviour varied greatly above and below the weir, where individuals in the more lentic upstream environment nested in potentially sub-optimal habitats. Our results demonstrate the potential effects of low-head weirs on movement and behaviour of freshwater fishes.

## Introduction

Globally there has been a huge proliferation of barriers in aquatic environments^1^. For society, in-stream barriers facilitate transportation, water supply, flood control, agriculture and power-generation^2^, but this all comes at the expense of riverine ecosystems. These barriers also prevent many species completing important stages of their life-histories^3–5^. Whilst there is much focus on large instream structures, such as dams, low-head weirs that periodically drown out can also significantly alter aquatic habitat by changing river hydraulics^6^ and by acting as a significant barrier to movement^7,8^, particularly if drown-out events are short-lived and/or unpredictable.

Water infrastructure creates physical barriers to longitudinal connectivity, affecting the ability of fish to move between different habitats to complete their life-history requirements^5^. These physical barriers can impede broad-scale migrations, preventing amphidromous fish from reaching spawning grounds and diadromous fish returning upstream to suitable habitat^9^, they can also bring about changes in species assemblage structure^10^ and have also been found to affect the genetic structure in potamodromous fish populations^8^. The physical barrier presented by a dam or weir can also interfere with short-term, localized movements among habitats which is important for foraging^11^, escape from predators^12,13^ and access to thermal refugia^14–16^.

In addition to the physical impediment posed by weirs and dams, the hydraulic environment immediately up- and downstream of weirs can also be often significantly altered. As environmental variations often cue and guide fish movements^17–19^, these zones can form ecological barriers. An ecological barrier is formed immediately upstream of physical barrier, where the hydraulic characteristics (e.g. water velocity) that stimulate and help orient fish movements are altered^20^. In this case, habitats become lentic and may be more akin to non-riverine, lacustrine environments, which can affect fish behaviours at the local-scale^14^. Alteration of upstream hydraulic conditions leads to habitat loss and changes in water velocity and temperature^20^, all of which are important factors influencing fish habitat use^21^, foraging^22^ and reproduction^23^.

While there have been many studies focussing on the effects of barriers on broad-scale movements^24^, and to a lesser degree, low resolution studies of local-scale movements^14^, much less is known about how weirs alter local habitat use and movement patterns. Similarly, little is also known of how a barrier may obstruct, or how a reservoir may affect routine fish movements at a local-scale. These studies have only investigated position of fish in relation to the barrier and distance of movements^24^, excluding metrics of movement such as orientation or rate of movement (ROM). Considering these aspects of movement may offer insights on how barriers affect fundamental components of fish life histories, such as foraging behaviours or energetic expenditure. The physical barrier of dams to reproductive migrations is well established^25^, however the ecological effect of instream barriers on reproduction has seldom been considered^26^. The reservoir created by a barrier may alter or delay abiotic cues, such as water temperature or river discharge, to reproductive behaviours in fish, and may also alter suitable nesting habitat for some species.

This study focuses on the movement behaviour of three species of freshwater fish: eastern freshwater cod (*Maccullochella ikei*), freshwater catfish (*Tandanus tandanus*), and Australian bass (*Percalates novemaculeata*). All three species are endemic to eastern Australia, with *M. ikei* and *P. novemaculeata* only occurring in coastal rivers, while the range of *T. tandanus* extends inland to include the Murray-Darling Basin. *Maccullochella ikei* is a nationally listed threatened species^27^ and has undergone a dramatic decline in population size since European settlement, due in part to habitat alteration and river fragmentation^28^. *Tandanus tandanus* has declined throughout much of its range and this species is currently listed as Endangered^29^. Concerns have also been expressed about effects of instream barriers and hydrological alteration on *P. novemaculeata* populations^30,31^. Relatively little is known about the movement biology of these species. *Tandanus tandanus* and *M. ikei* both typically show limited movement^32–34^, however *M. ikei* have been recorded making occasional long-distance movements in response to increased river discharge^34^. Neither *M. ikei* or *T. tandanus* are known to make large-scale breeding movements; instead *M. ikei* establish local spawning sites and deposit eggs under large boulders of bedrock shelves between August and October^35^, while *T. tandanus* form nests of specifically arranged substrate in open water between October and January^36,37^. *Percalates novemaculeata* are a catadromous species that make large-scale breeding migrations downstream to estuarine reaches in response to elevated winter flows, before returning to freshwater reaches in spring^38^.

This study aimed to quantify the effects of a low-head weir on multi-scaled movement and behaviour of *M. ikei*, *P. novemaculeata* and *T. tandanus* in the Nymboida River, eastern coastal Australia. Whilst the impacts of large dams are well studied, the physical and ecological effects of low-head weirs that are periodically drowned out by high flows are less well-understood. With three native species of distinct movement behaviours, we used acoustic telemetry to evaluate differences in the longitudinal movements upstream and downstream of a low-head weir and quantify activity (ROM) and spatial occupancy within the weir pool over four months. In addition, we compared nesting behaviours of *T. tandanus* up- and downstream of the weir. We hypothesized that fish movement behaviours between reaches above and below the weir would differ, at both broad- and fine-scales, with the more mobile *P. novemaculeata* showing the greatest difference. We also hypothesised that the nest-site selection of *T. tandanus* would vary between the environments up- and downstream of the weir. This paper presents a detailed study into a poorly understood, and seldom researched, facet of the impacts of instream barriers.

## Methods

### Study area

**Figure 1:**
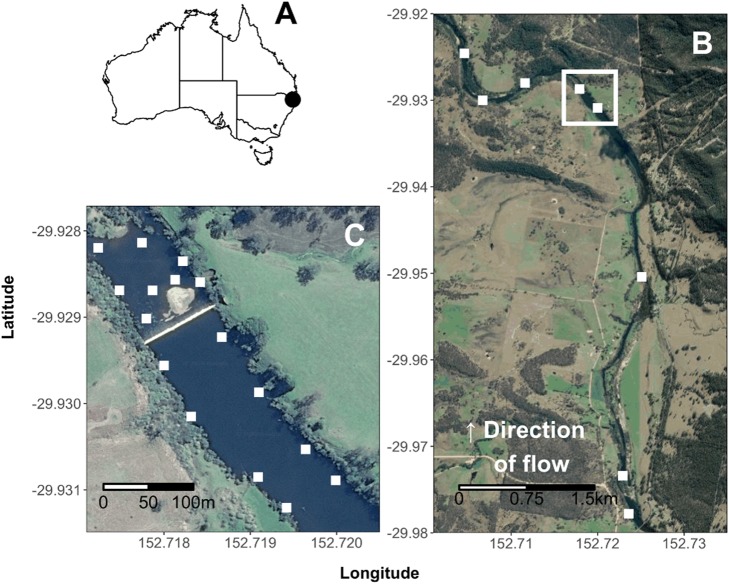
(**A**) Location of Nymboida River in eastern Australia, (**B**) locations of acoustic telemetry receivers in the linear array in the Nymboida River surrounding Nymboida Weir (VPS array and weir within box), and (**C**) locations of receivers in the fine-scale VPS array upstream and downstream of Nymboida Weir. Map data: Google, Maxar Technologies, USA.

**Figure 2:**
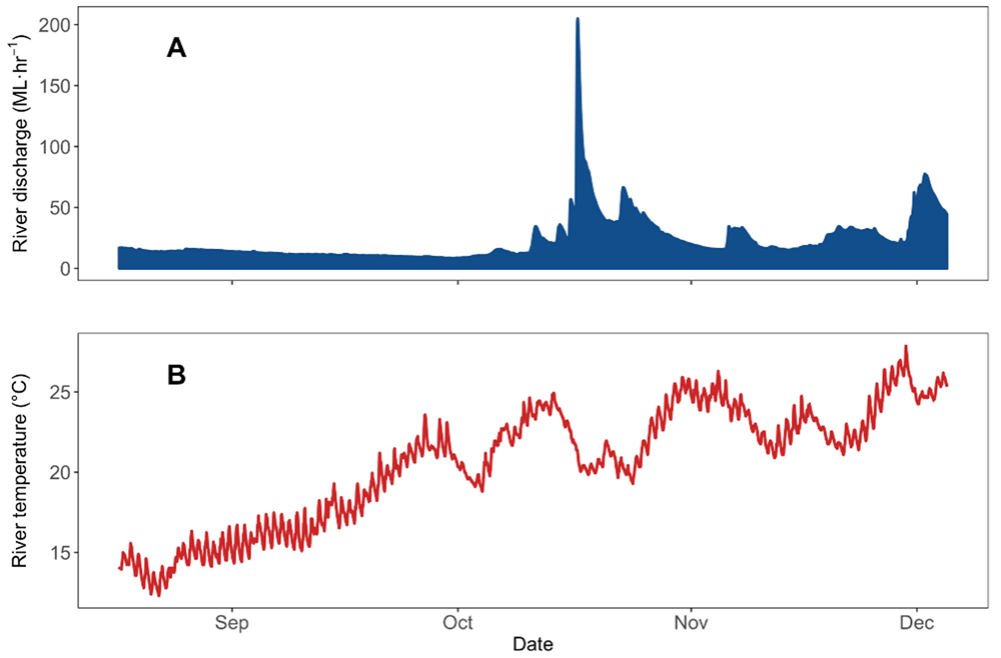
(**A**) Nymboida River discharge (ML·h^−1^) and (**B**) water temperature (°C) for the study period.

The Nymboida River is a largely unregulated, perennial river located in the Clarence River Basin, NSW, Australia (Fig. 1a), and has a catchment area of 1732 km^2^. The study reach was located either side of Nymboida Weir, the only artificial barrier in the river (Fig. 1b,c), which is approximately 50 km above the confluence of the Mann and Nymboida rivers (GPS coordinates 29.929086°S, 152.718160°E). The weir is a of a rectangular concrete design, 4.26 m in height and has no fish passage infrastructure. Water flowed over the weir for the entire duration of the study (August 2017–January 2018). The study period and duration were selected to coincide with the *T. tandanus nesting* period, while minimizing the risk of damage to the fine-scale VPS array by potential large summer flow events. Stream width varied between 30 m and 95 m, and 1 and 4 m in depth within the study reach. River discharge during the study mostly remained <50 ML·h^−1^, however it received one small flow pulse (204 ML·h^−1^ equivalent to a ~95^th^ percentile instantaneous flow event), which was not large enough for weir drown-out (Fig. 2a), as well as several smaller increases in flow. Water temperature ranged from 12.3 to 27.9 °C (Fig. 2b).

### Linear acoustic telemetry array

Broad-scale fish movements were recorded from 14 August – 05 December 2017 using an extensive linear array of eight Vemco VR2W 69 KHz receivers. These were deployed at varying intervals (<3 km apart) along the Nymboida River to ensure each reach would have reception (Fig. 1b), with four receivers situated above the weir and five below the Nymboida Weir. The broad-scale receiver array recorded binary presence/absence data when a tagged fish entered the reception range (maximum range ~ 400 m) of a given receiver. A temperature logger (OneTemp, Sydney) was also deployed at the receiver location situated immediately upstream of the weir. River discharge recorded at Water NSW gauge #204001, 4 km upstream of the linear array.

### Fine-scale acoustic telemetry array

A Vemco Positioning System (VPS) acoustic array was deployed in 400 m of river immediately upstream and downstream of the weir, respectively, from 14 August–05 December 2017. Upstream of the weir, eight Vemco VR2W 69 KHz receivers were deployed in a grid, at intervals approximately equal to the river width (Fig. 1c). Another eight receivers were deployed downstream of the weir but in equilateral triangles, so as to better accommodate the more complicated river morphology. A series of range tests were performed as per Espinoza *et al*.^39^ *in situ* to assess signal strength and to determine the maximum distances receivers could be spaced apart so as to allow simultaneous high precision positioning of multiple fish. The reception range of receivers in the fine-scale array was similar to those in the broad-scale array.

### Fish collection, surgical tagging and recording

A total of 34 adult fish were collected, tagged and released over two trips on 28 July 2016 and between 26–27 July 2017 (Table 1, see Appendix 1 for full details of fish tagged and detected during the study). Fish were collected using a boat-mounted electrofisher (2.5 GPP unit; Smith-Root, WA, USA) and were kept in a submerged cage until the tagging procedure. Upstream of the weir wall, fish were collected, tagged and released within the detection area of the fine-scale array. Downstream of the weir wall, fish were collected, tagged and released ~200 m downstream of the fine-scale array, due to difficulties accessing this part of the river by boat.

**Table 1:** Number of fishes tagged and location of capture and release in relation to weir in the Nymboida River.

	Upstream		Downstream	
	2016	2017	2016	2017
*M. ikei*	1	5	2	5
*P. novemaculeata*	3	5	—	2
*T. tandanus*	—	5	—	6

Fish were anaesthetised in river water containing 50 mg·L^−1^ benzocaine (ethyl-*p*-aminobenzoate; Sigma Aldrich, Shanghai, China) and weighed (g) and measured (mm). Fish were then transferred to an operating cradle, with water containing an equivalent level of anaesthetic (50 mg L^−1^) continually pumped over the gills to maintain anaesthesia. To access the peritoneal cavity, a small incision was made through the body wall of the fish, adjacent to the linea alba and anterior of the anal vent. The gonads of the fish were examined through the incision to determine sex before the insertion of an acoustic tag. Either a Vemco V9 or V13 69 KHz acoustic telemetry transmitter tag was used for each fish to keep below the recommended 2.25% of body weight^17,40,41^. A passive integrated transponder (PIT) tag was also inserted in the cavity. The incision was closed with two or three sutures using 0.3 mm pseudo-monofilament, non-absorbable thread (Vetafil Bengen; WdT, Garbsen, Germany). After suturing, the fish was given an intramuscular oxytetracycline hydrochloride (0.25 mL kg^−1^) injection (CCD Animal Health and Nutrition, Toowoomba, Australia) to inhibit infection and returned to the submerged cage to recover. All studies were performed in accordance with the relevant guidelines and regulations, reviewed and approved by the NSW DPI^29^ Animal Care and Ethics Committee (permit numbers 06/06 and 98/14).

After 112 days all receivers were downloaded and uploaded into a purpose-built database. Data from the VPS array was sent to the VPS service (Vemco, Halifax) for processing, whereby positions were generated by measuring the differences in transmission detection times at several different time-synchronized receivers.

### Nest site surveys

To evaluate the potential effects of the Nymboida Weir on the nesting timing and nesting habitat selection of *T. tandanus*, periodic visual surveys of nest locations were undertaken 450 m up- and downstream of the weir. Nest surveys were performed weekly to fortnightly, depending on weather conditions, during the known reproductive period^37,42^ from 11 September–16 December 2017, with a final survey undertaken on 19 January 2018. Nest surveys were conducted along four longitudinal transects within both study reaches in a boat, with each transect equally spaced across the river width. Nests present in less than 2 m depth were located by direct observation, while all areas of >2 m depth were inspected with an underwater camera (SeaViewer, FL, USA) and 10″ LCD monitor. Water clarity during the surveys was mostly high (secchi depth ~1.8 m), but on occasions when clarity was low, the underwater camera was employed in any areas where the river bed was not visible from the surface.

*Tandanus tandanus* nests are generally characterised by a large (0.5 to >2 m diameter) circular depression in the substrate, composed of carefully arranged coarse gravel and cobble, encompassed by a fine gravel or sand ring^37,43^. Nests were recorded as either forming, active or abandoned. Forming nests were defined as having an incomplete, patchy ring structure and no fish present, active nests possessed a complete ring structure with clean substrate, and abandoned nests possessed uncleaned substrate with silt build-up and no fish present.

Nest site characteristics were recorded at the centre of each nest located. A handheld GPS unit (Garmin, KS, USA) was used to record the position. Water depth was measured using a portable depth sounder (Honda Electronics Co., Aichi, Japan). Water velocity was recorded at 10, 60 and 90% of total depth with a portable flow meter (Swoffer, WA, USA). Riparian vegetation canopy cover was estimated using a spherical densiometer. Substrate composition (same classes as habitat assessment) was visually estimated from the surface separately for both the nest and 1 m surrounding the nest. Nest diameter was recorded using a plastic bar marked at 100 mm intervals which was positioned immediately above the nest. Fish presence and mesohabitat type (riffle, run or pool) was also recorded.

### Habitat assessment

Aquatic habitat characteristics were quantified for river 450 m up- and downstream of the weir and used to relate to nest site selection of *T. tandanus*. Habitat assessment was conducted using a modified version of the in-line transect method^44,45^. Transects were taken perpendicular to stream flow and were spaced within each study reach at distances of ~4% of the total reach length of 450 m (25 transects in total, 18 m apart). The first and last transects were placed at a distance of 0% and 100% of the total reach length.

Habitat characteristics were recorded at six points along each lateral transect, situated equidistantly and beginning and ending at each bank. At each point, a handheld GPS unit (Garmin, KS, USA) was used to record the position. Water depth was measured using a portable depth sounder (Honda Electronics Co., Aichi, Japan). Mean water velocity was recorded with a portable flow meter (Swoffer, WA, USA). The presences of substrate types (mud, sand, fine gravel, coarse gravel, cobble, rock and bedrock) and submerged physical structures (aquatic macrophytes, submerged marginal vegetation, submerged overhanging vegetation, emergent vegetation, root masses, undercut banks, large wood and small wood) characteristics were visually estimated for 0.5 m^2^ around each sample point. Riparian vegetation canopy cover was estimated using a spherical densiometer.

### Data analysis

#### Broad-scale movement behaviour

The distance of each acoustic receiver location from the most downstream receiver was calculated by digitising satellite images of the study reach into a spatial object in ArcGIS 10.4 (ESRI, Redlands, CA, USA). The spatial object was then converted into a distance matrix in the V-Track package^46^ in R (R Development Core Team; www.r-project.org). Individual fish detections were then matched with the distance matrix and the distance between detections was calculated. Data on river discharge, water temperature and diel period (calculated with the *insol* package^47^) was assembled for each hourly increment over the duration of the study.

Variation in broad-scale fish movement in relation to the instream barrier and environmental variations were investigated using a hurdle mixed model using the *lme4* package in R^48^. As the data were of a continuous nature and was zero-inflated, we used a model that specified a logistic regression (GLMM) component for the binary response (presence or absence of movement), and then a standard linear mixed model (LMM) for the continuous non-zero response (distance moved). In this model, the distance travelled since last detection (maximum for each hour) was the response variable and position in relation to weir (upstream or downstream), hourly river discharge, water temperature, diel period, lunar phase and body length were the independent variables. Fish identity (ID) was included as a random effect in the model. Using Akaike information criterion (AIC), all possible models and interactions were examined, and the best model was selected. Following the protocol of Zuur *et al*.^49^, we check for statistical outliers and collinearity among predictor variables was assessed using variance inflation factors (VIF) in R. Model residuals were checked for normality and model fit was assessed by comparing model residuals and fitted values. A model was fitted only for *P. novemaculeata* too few movement events were recorded for *M. ikei* and *T. tandanus*.

#### Fine-scale movement behaviour

Fish presence and absence within the fine-scale arrays were examined and plotted to determine possible transmitter errors and to identify which individuals to include in the analysis. Due to the low number of fish detections in the fine-scale array downstream of the weir, this area was excluded from subsequent analysis. All detections from downstream fine-scale array receivers were, however, included in the broad-scale movement analysis. Prior to analyses, data were filtered by horizontal position error (HPE), which is a relative measure of horizontal error sensitivity^39^. Only locations with an HPE < 7 were included for analyses. Generally, these HPE thresholds corresponded to positional errors of <5 m (3.03 ± 0.567 m, mean ± s.e.) when examining errors of static tags placed within the array^39,50^.

Fine-scale movement activity in the weir pool was investigated using rate of movement (ROM) expressed in m·s^−1^. Average hourly ROM was generated as a measure of fish activity using the *adehabitatLT* package^51^ in R. This was calculated by dividing the step length between two consecutive fish locations by the time taken between the two positions (expressed as m·min^−1^). The maximum delay between transmissions of the tags used in this study was of 160 seconds. To avoid underestimating ROM, only consecutive positions less than 160 seconds apart (the maximum time between transmitter pings) were included in this analysis^50^. Each movement between detections was also categorized by orientation; either longitudinal (upstream and downstream), or lateral (bank to bank). For the study reach within the fine-scale array, downstream orientation ranged between 310° to 350° and upstream from 130° to 170°, and all other orientations were considered lateral.

A GLMM with a gamma distribution and a log-link was used to fit hourly fish ROM data in the *lme4* package in R^48^. The initial model included ROM as a response variable and river discharge, water temperature, orientation of movement, diel period, lunar phase and body length as independent variables. Fish ID was included as a random effect in the model. Using AIC, all possible models and interactions were examined, and the best model was selected. In the same manner as the broad-scale analysis, we followed the protocol of Zuur *et al*.^49^ to satisfy model assumptions. Each species was analyzed separately.

Patterns of spatial occupancy for each species was examined using kernel utilization distributions (KUD) in the *adehabitatHR* package in R^51^. For each fish, a 95% and 50% KUD was produced from VPS positions, after HPE filtering, and was visually examined for spatial and temporal trends. KUDs were not generated for fish with less than 500 detections.

#### Nesting behaviour of T. tandanus

*Tandanus tandanus* nest site selection in relation to habitat availability was evaluated using Euclidean distance-based analysis (EDA) as per Conner and Plowman^52^. This method was chosen as even if errors occur in habitat assignment, the distance to the correct habitat is reduced^52,53^. Firstly, each discrete habitat type recorded in the study area were interpolated using universal kriging from sampled transect points within ArcGIS 10.4 Geospatial Analyst extension (ESRI, California). Within each upstream and downstream study reaches, 1000 random points were generated and distances between each habitat type and each random point were calculated. A vector of mean distances to each habitat type was then created. For each nest site, the distance to all habitat types was also determined (distance to the habitat occupied by the nest was 0). EDA ratios were calculated as the mean observed distance (from nest sites) divided by the mean expected distance (from random points) to each habitat type. A unique EDA ratio was calculated for each nest site, retaining the individual nest site as the experimental unit. If habitat use is random, all EDA ratios should equal one, with values >1 indicating positions farther from a habitat type than expected (avoidance) and values <1 indicating positions closer to a habitat type than expected (preference). Nest occurrence over time and duration were also plotted and compared between upstream and below the weir.

## Results

### Broad-scale movement

**Figure 3:**
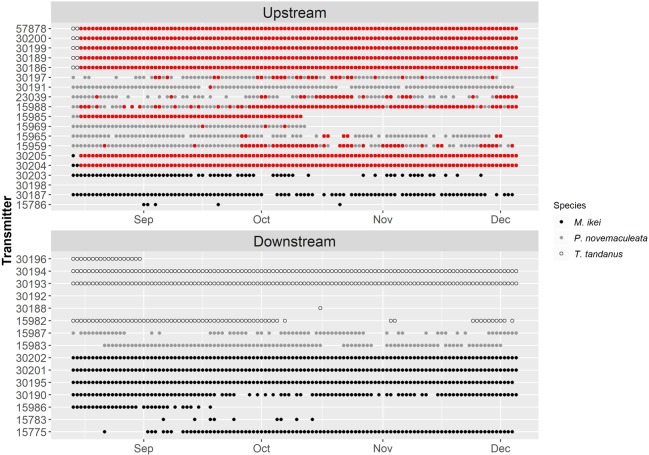
Days detected in the linear array in the Nymboida River upstream and downstream of the Nymboida Weir for *M. ikei, P. novemaculeata* and *T. tandanus*, over the total study duration of 112 days. Each dot represents at least one detection in a given day. • denotes at least one detection within the fine-scale VPS array within the Nymboida Weir pool on a given day.

With the exception of *T. tandanus* ID30192, all fish released into the downstream linear array were detected at least once (Fig. 3). All fish except *M. ikei* ID 30198 released in the upstream linear array were detected at least once (Fig. 3).

In the downstream linear array, *M. ikei* were detected on average for 81.43 ± 16.37 (S.E.) days, *P. novemaculeata* were detected 78.5 ± 13.5 days and *T. tandanus* were detected for 52.33 ± 21.79 days (Fig. 3). In the upstream linear array, *M. ikei* were detected for the 66.17 ± 21.16 days, *P. novemaculeata* were detected 87.12 ± 7.34 days and *T. tandanus* were detected for 112.6 ± 0.25 days (Fig. 3).

**Figure 4:**
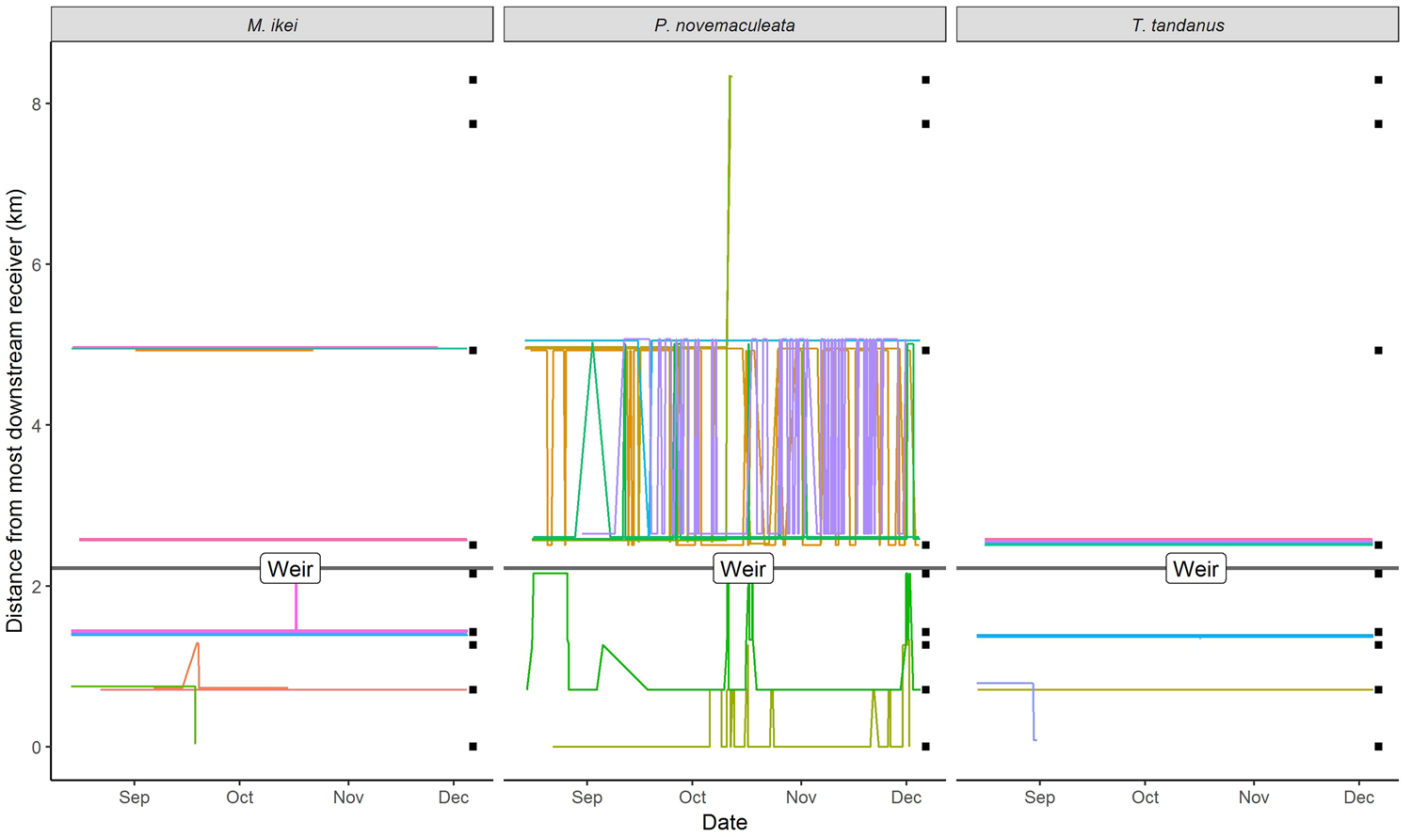
Hourly movement trajectories of all tagged fish detected within the broad-scale linear array in the Nymboida River over the study period. Lines show distance moved relative to the most downstream receiver with each fish ID represented by a different coloured line and individuals from each species grouped by panel. Black squares on right denote acoustic receiver positions. The location of the Nymboida Weir is indicated with a black horizontal line and the detection area of the fine-scale VPS array within Nymboida Weir pool is indicated with grey shading.

No individuals of any species crossed the weir over the study period (Fig. 4). *Maccullochella ikei* and *T. tandanus* were relatively sedentary. Upstream of the weir wall, most fish released within the weir pool remained there for the entire study period (Fig. 4). The exception to this was for three *M. ikei* that spent the majority of time ~3 km further upstream. Downstream of the weir, three *M. ikei* and one *T. tandanus* undertook movements between broad-scale receivers, with two *M. ikei* making short upstream return movements, and one *M. ikei* and one *T*. *tandanus* moving downstream and out of the detection range of the array.

*Percalates novemaculeata* was considerably more mobile than the other two species (Fig. 4). Upstream of the weir, most individuals made numerous repeated longitudinal movements between the weir wall and the receiver situated approximately 3 km upstream. *Percalates novemaculeata* ID 30197 was particularly mobile, making 65 movements over the 112 days of study. One of the two *P. novemaculeata* released downstream of the weir (ID 15987), made six upstream movements terminating at the weir wall before returning downstream (Fig. 4). For all but three fish, all upstream or downstream movements were interrupted by the weir (Fig. 4).

The location of individual *P. novemaculeata* relative to the weir (upstream of downstream) had no significant effect on probability of fish movement, however fish located above the weir moved significantly greater distances when they did move (>2 km on average; Table 2). Increases in both river discharge and temperature were significantly associated with movement events, but had no effect on distance moved (Table 2). A significant interaction between weir position and temperature indicated that fish located upstream of the weir were less likely to move at lower temperatures, and moved shorter distances when they did (Table 2).

**Table 2:** Coefficients of hurdle linear mixed model relating environmental variables to the probability (presence-absence component) and distance (non-zero component) of broad-scale movements of *P. novemaculeata* in the Nymboida River.

Fixed effects	Presence-absence component (Binomial)		Non-zero component (Gaussian)	
	Estimate ± std. error	*P*	Estimate ± std. error	*P*
Weir position - Upstream	2.84 ± 1.53	0.06	2328 ± 314	**<0.01**
River discharge	0.03 ± 0.01	**<0.01**		
River temperature	0.19 ± 0.05	**<0.01**	20 ± 11	0.08
River discharge: Upstream	-0.01 ± 0.01	0.06		
River temperature: Upstream	−0.13 ± 0.06	**0.04**	−28 ± 13	**0.04**
**Random effect (fish ID)**				
N (fish ID)	9		9	
Std. Dev.	0.75		80.84	
Observations	6489		178	

#### Fine-scale movement

In the fine-scale array upstream of the Nymboida Weir, all tagged *T. tandanus* and *P. novemaculeata* were detected at least once during the study period (Fig. 3). Three of the five *M. ikei* tagged were not detected within the array. All five *T. tandanus* were detected for the entire 112 days (Fig. 3), while *M. ikei* and *P. novemaculeata* were detected within the array for 44.8 ± 27.43 and 30.5 ± 9.78 days (mean ± standard error), respectively (Fig. 3).

**Figure 5:**
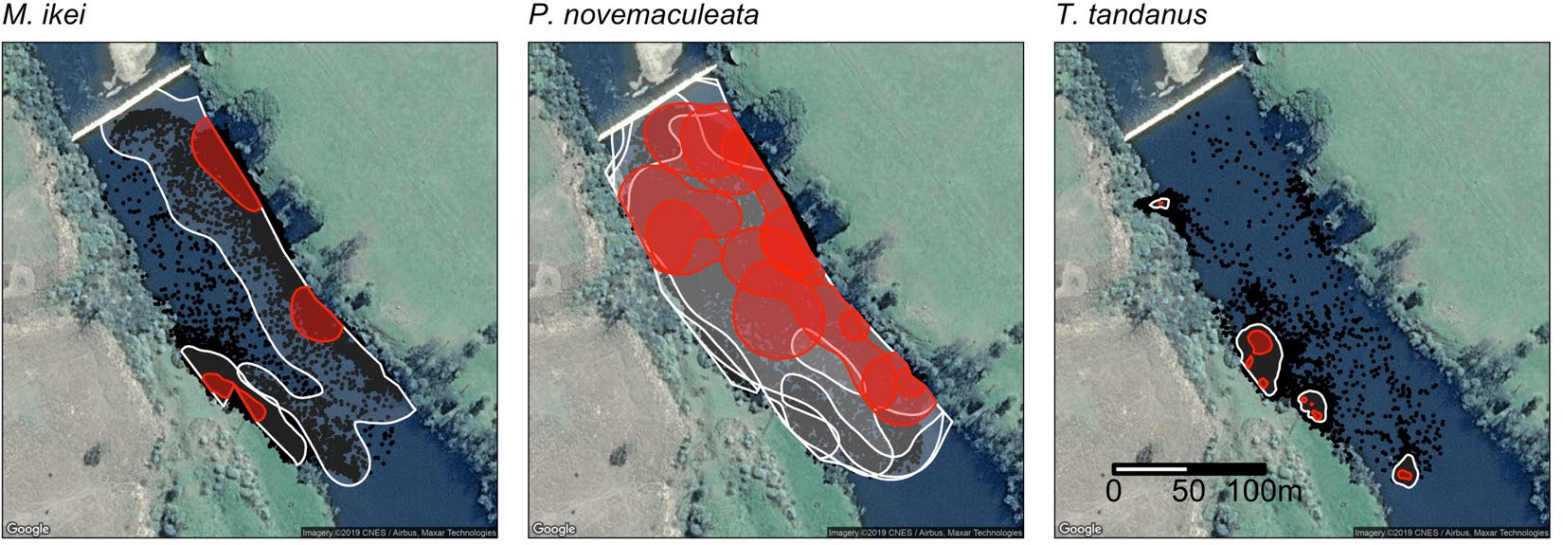
Spatial occupancies (50% and 95% KUD) for *M. ikei* (n = 2, Fish ID 30204 and 30205), *P. novemaculeata* (n = 4, Fish ID 15959, 15985, 15988 and 23039) and *T. tandanus* (n = 4, Fish ID 30186, 30189, 30200 and 57878) in the fine-scale array in the Nymboida Weir pool. Raw detections for each species are denoted by black points. Map data: Google, Maxar Technologies, USA.

Spatial occupancy KUDs showed a high level of interspecific variation (Fig. 5). Individuals of *P. novemaculeata* had the largest home ranges, often making use of the entire weir pool, whereas individuals of *M. ikei* and *T. tandanus* had much smaller home ranges sizes concentrated closer to the river banks. Both *P. novemaculeata* and *M. ikei* were frequently detected right against the weir wall, while *T. tandanus* was rarely detected close to the weir wall.

**Figure 6:**
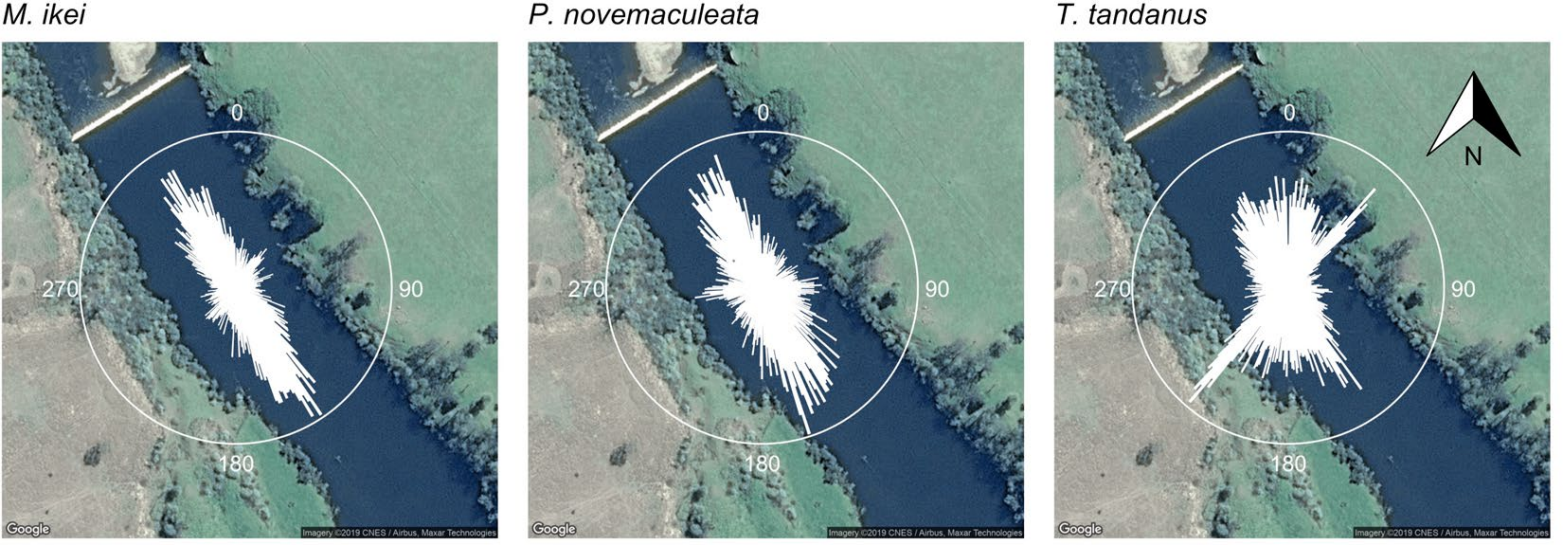
Circular histograms of relative frequency of directions (azimuth) of movements for (**A)** *M. ikei*, (**B**) *P. novemaculeata* and (**C**) *T. tandanus* within the Nymboida Weir pool. Bars that reach closer to the circular border are of higher relative frequency, and those that are closer to the centre are of lower. Histogram bars are binned at 1 °. Only individual movement events >10 m within 160 seconds between detections are included. Map data: Google, Maxar Technologies, USA.

Regression analyses of fine-scale movement behaviour in response to environmental variation revealed that all three species were more active in the longitudinal orientation than the lateral orientation and were significantly less active at higher river discharge (Fig. 6; Table 3). Both *M. ikei* and *T. tandanus* were more likely to move in a longitudinal orientation when river discharge was higher, while river discharge had no effect on *P. novemaculeata* activity orientation (Table 3). *Percalates novemaculeata* was significantly less active during the day, while *M. ikei* was most active during the dawn period and *T. tandanus* was significantly less active during the day and dusk periods (Table 3). *P. novemaculeata* exhibited significantly higher longitudinal activity during the night (Supplementary material 1; Table 3), *M. ikei* showed significantly higher longitudinal activity during the day and dusk periods (Supplementary material 1; Table 3) and *T. tandanus* showed little longitudinal activity, but when they did move it was mostly in the dawn period (Supplementary material 1; Table 3).

**Table 3:** Coefficients of generalized linear mixed model (family = Gamma (*log-link*)) relating environmental variables to fish activity (Rate of Movement; m·s^−1^) within the Nymboida River fine-scale array.

Fixed effects	*M. ikei*		*P. novemaculeata*		*T. tandanus*	
	Estimate ± std. error	P	Estimate ± std. error	P	Estimate ± std. error	P
River discharge	−0.01 ± 0.01	**<0.01**	−0.01 ± 0.01	**<0.01**	−0.01 ± 0.01	**<0.01**
Longitudinal movement	0.33 ± 0.04	**<0.01**	0.34 ± 0.05	**<0.01**	0.3 ± 0.02	**<0.01**
Day	−0.71 ± 0.03	**<0.01**	−0.23 ± 0.03	**<0.01**	−0.05 ± 0.01	**<0.01**
Dusk	−0.44 ± 0.03	**<0.01**	0.21 ± 0.04	**<0.01**	−0.09 ± 0.02	**<0.01**
Night	−0.15 ± 0.03	**<0.01**	−0.04 ± 0.03	0.23	0.01 ± 0.01	0.99
River dis.: longitudinal.	0.01 ± 0.01	**<0.01**			0.01 ± 0.01	**0.01**
Day: longitudinal	0.21 ± 0.05	**<0.01**	−0.09 ± 0.05	0.1	−0.06 ± 0.03	**0.02**
Dusk: longitudinal	0.15 ± 0.06	**0.01**	−0.07 ± 0.07	0.35	−0.08 ± 0.03	**0.01**
Night: longitudinal	−0.16 ± 0.04	**<0.01**	0.18 ± 0.06	**0.01**	−0.07 ± 0.03	**0.01**
**Random effect (fish ID)**						
N (fish ID)	2		7		5	
Std. Dev.	0.02		0.18		0.67	
Observations	32970		19222		142704	

#### Nesting behaviour of *T. tandanus*

**Figure 7:**
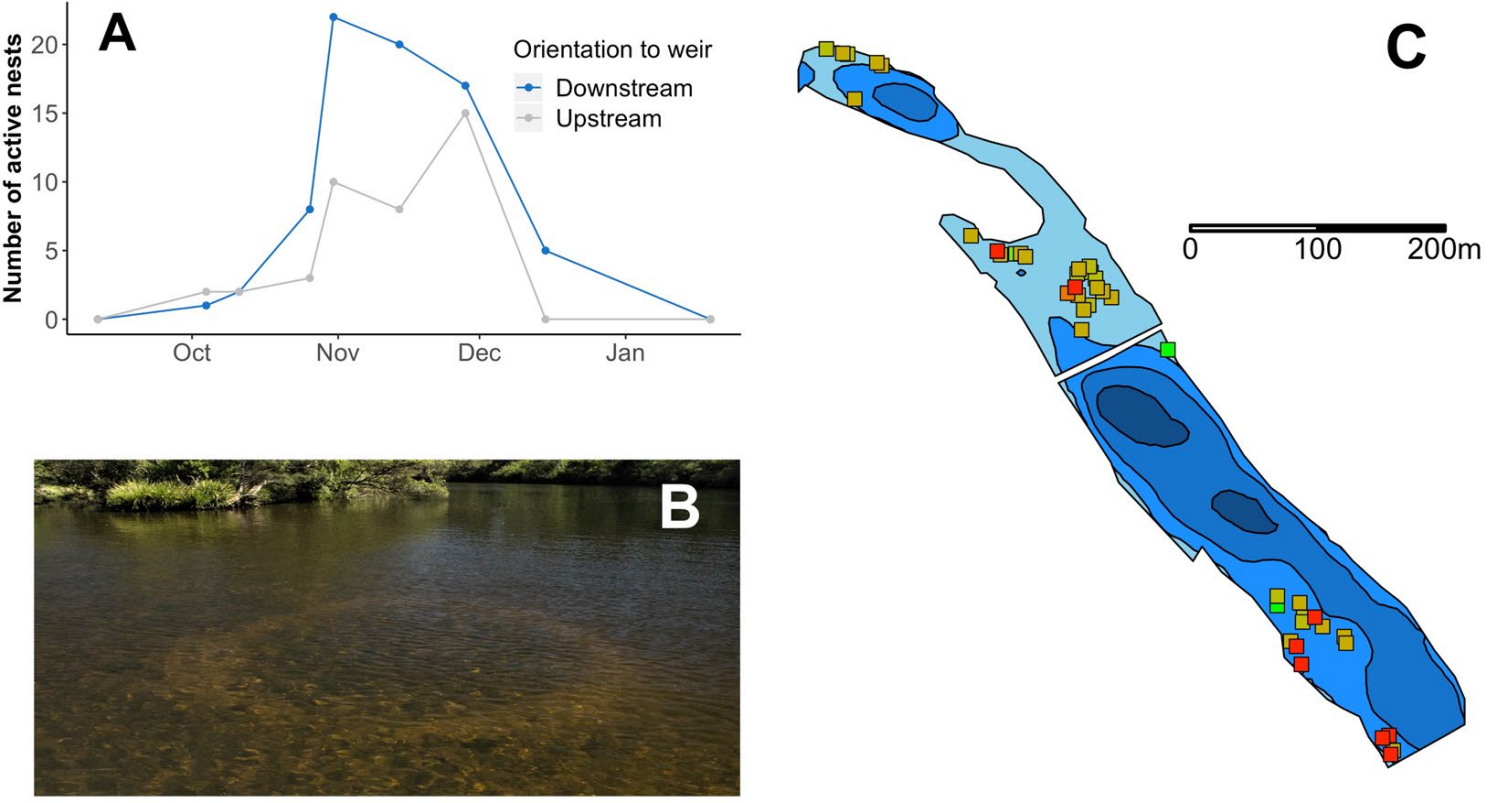
(**A**) Temporal variation in frequency of active *T. tandanus* nests in the Nymboida River above and below the Nymboida Weir. (**B**) Photograph of active *T. tandanus* nest (1.8 m diameter) in 0.8 m of water. (**C**) Location of *T. tandanus* nests site in the Nymboida River (Nymboida Weir visible as white bar). Nest sites are coloured by timing of occurrence, with earliest nests denoted by green, through to latest nests denoted in red. Depth contours shown in intervals of 1 m, beginning at 0–1 m in light blue, and ending at 3–4 m in dark blue.

Forty-four *T. tandanus* nests were observed throughout the 450 m up- and downstream of the weir during the study period (Fig. 7). Nests were first observed forming on 4^th^ October 2017, shortly after water temperature first reached 24 °C, and were last observed as being active on 12^th^ December 2017 (Figs. 7a,c and 2b). Upstream of the weir, the number of nests gradually increased in frequency until late October, when several nests were abandoned, and then increased again to reach a peak of 15 active nests in late November. Active nests decreased rapidly thereafter and disappeared completely by mid-December. Downstream of the weir, nests increased consistently to a peak frequency of 21 active nests in late October, after which they gradually declined, disappearing completely by mid-January. Nesting duration varied among individuals and location in relation to the weir; nests located upstream lasted for a shorter duration than those located downstream (mean ± s.e. 20 ± 5 days versus 29 ± 4 days, respectively). Regardless of the timing of individual nest formation, most remained active (Fig. 7b) until late October when most nests were abandoned. Nests were present throughout the area immediately below the weir, however, except for one that was abandoned early in the study, no nests occurred within the deep impounded area up to 400 m upstream of the weir wall (Fig. 7c). Nests were recorded at a maximum water column depth of 2 m. Both upstream and downstream of the weir, many fish were observed occupying two nests within close proximity, and when disturbed from one nest, would relocate to the other. In one case, two nests were joined into one larger nest throughout the nesting period.

**Figure 8:**
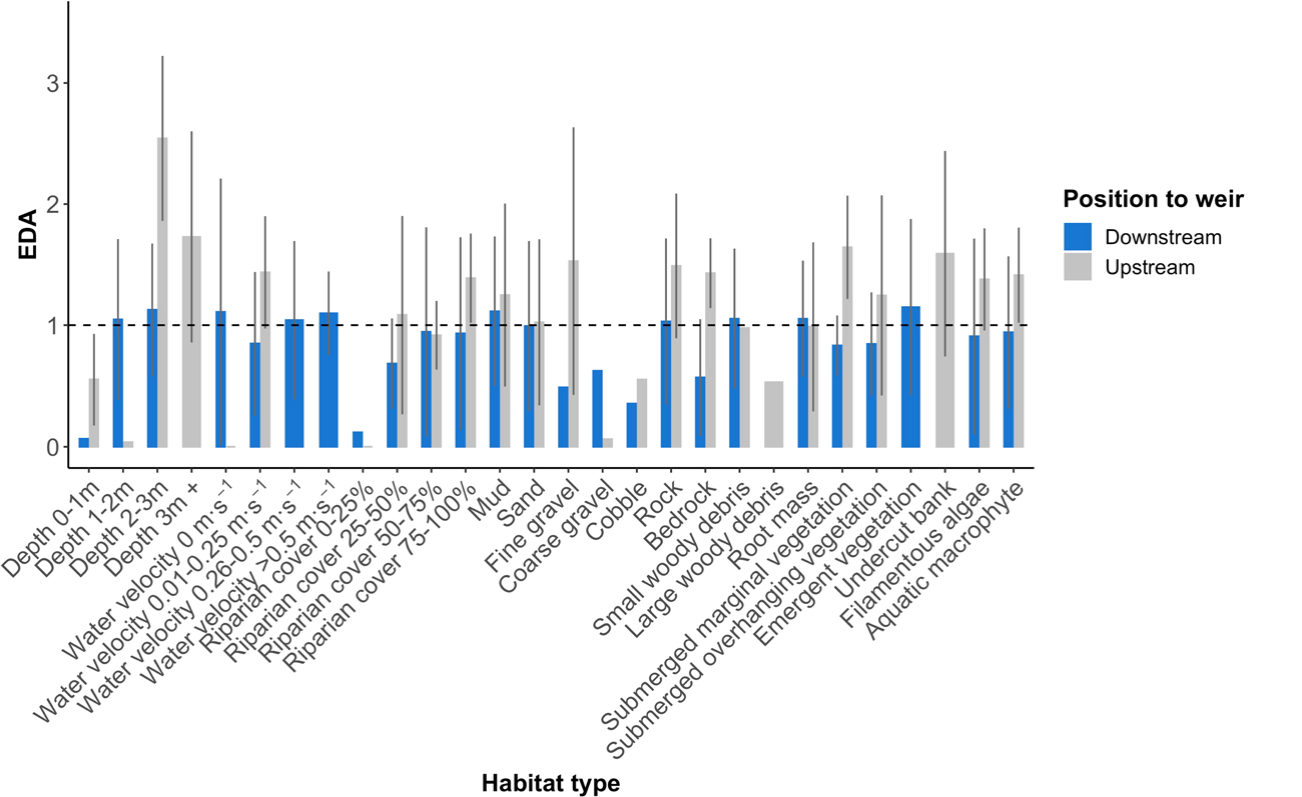
Habitat selection of *T. tandanus* nests above and below the weir in the Nymboida River. Habitat type selection was based on Euclidean distance analysis (EDA) ratios of microhabitat types available. Values <1 indicate increased use relative to availability, while values >1 indicate avoidance.

Analysis of habitat characteristics of *T. tandanus* nest sites based on EDA ratios indicated non-random nest site selection for both upstream and downstream fish (Fig. 8). *Tandanus tandanus* preferred nest sites in areas with coarse gravel and cobble substrates and low riparian canopy cover (<25%) with little difference between upstream and downstream areas. Upstream fish selected deeper areas with lower water velocities for nesting compared with downstream fish (Fig. 8). Downstream fish also tended to select nest sites closer to submerged riparian and aquatic vegetation compared to upstream fish (Fig. 8).

## Discussion

This study aimed to quantify the effect of a relatively small instream barrier on local and broad-scale movements of three fish species and the nesting behaviour of *T. tandanus* in a largely unregulated river. While we were unable to provide a comparison of fish movements between riverine habitats with and without a weir, we were able to study longitudinal fish movement behaviours in relation to the barrier effect of the weir, and compare fish movement behaviours between the lentic environment within the weir pool, and more natural, lotic environments downstream of the weir. We found that none of the fish included in this study traversed the weir, however, many individuals upstream of the weir made multiple relatively large, longitudinal movements that terminated at the weir wall. We also found that the weir is likely acting as a strong physical and ecological barrier, affecting the broad-scale movement and reproductive behaviour of fish.

### Linear movement

Our findings indicate that the Nymboida weir is a physical barrier to fish movement, at least under the conditions experienced over the study period. Weirs without a fishway, as is the case at the Nymboida Weir, are known to prevent upstream fish passage during periods of low and even moderate flows^54,55^. Although still possible in the absence of a fishway, we recorded no downstream passage over the weir, and the occurrence of several small flow events during the study period were insufficient to cause drown-out of the weir. Throughout the study, many fish made multiple movements that terminated at the weir, before moving back upstream or downstream. Presumably, these movements were obstructed by the weir, and it can be assumed the fish would have continued in their respective direction had the physical barrier not been present. Apart from potential obstruction of departing and returning migrations for catadromous species such as *P. novemaculeata*, the physical barrier to movement may be limiting access to refugia and resources for fish either side of the weir^11,13–15^. Limiting access to refugia and resources has been shown to lead to a reduction in fitness of individuals and populations across multiple taxa^56–58^. The majority of broad scale movements of *P. novemaculeata* appeared to terminate at the receiver approximately 3 km above the weir. This may be a slight underestimate of the extent of upstream movements as the next receiver was situated a further 2 km upstream and fish were not able to be detected between these receivers. The apparent termination of movements may also have been influenced by the fact that the weir pool has fully transitioned back to a more lotic environment at this point and the consequent change in hydraulic environment may have cued the fish to cease moving upstream.

Broad-scale movements were most prevalent in *P. novemaculeata* compared to the other two species. *Percalates novemaculeata* are known to make frequent upstream and downstream broad-scale movements, even outside of their breeding migration^19,38^, whereas *T. tandanus*^32,33^ and *M. ikei*^34^ occupy mostly limited ranges throughout the year. The weir appears to have had a strong ecological effect on *P. novemaculeata*, at least over the relatively short (5 month) duration of the study, with fish above the weir making significantly more broad-scale movements than those below (Fig. 4). The Nymboida Weir significantly altered the hydraulic characteristics of the river, creating a more lentic environment above the structure. Fish movements have been reported to vary relative to the size and shape of the water bodies in which they inhabit^59,60^. Gardner *et al*.^61^ found freshwater bream (*Abramis brama*) in a river containing a lock increased linear range sizes when water levels were raised and decreased linear movements when water levels were dropped. This may be related to the different energetic costs associated with lentic and lotic environments. Fish swimming in higher water velocities exert more energy than when swimming in lower water velocities^62^, suggesting that *P. novemaculeata* inhabiting the lentic environment above the weir would exert less energy than those in the lotic environment below. Also, riverine fish have been shown to seek areas of higher water velocity for increased foraging efficiency, due to the proportionally larger amounts of drifting invertebrate food^63^. *Percalates novemaculeata* above the weir would have to move more to seek such microhabitats, compared to those below. The behaviours displayed by *P. novemaculeata* in this study may not persist during particularly high flow events, however the conditions observed in this study are typical of the majority of the year, and as such *P. novemaculeata* would likely behave accordingly.

### Fine-scale movement

While we were unable to test our hypothesis of differences in fine-scale movement behaviours above and below the weir, the three species showed distinctive movement behaviours, varying in home range sizes, activity rates and movement orientation in the fine-scale array within the weir pool. In addition, movement behaviours varied significantly in response to flow conditions and diel period. These findings are consistent with the available knowledge of the movement ecology of these species^32,64^ and likely relates to interspecific differences in body form, microhabitat usage and feeding behaviours.

The mobile, non-benthic *P. novemaculeata* had the largest home ranges, often making use of the entire weir pool, whereas individuals of *M. ikei* and the benthic *T. tandanus* had much smaller home ranges sizes concentrated closer to the river banks. All species were also found to be most active in a longitudinal orientation, and both *M. ikei* and *T. tandanus* were more likely to reduce activity rates and move in a longitudinal orientation when river discharge was higher. This may be explained by these species avoiding high velocity areas in the mid channel during high discharge events and instead using edge-habitats^32,34^ with low water velocities, such as undercuts and marginal vegetation. The decrease in fine-scale activity as discharge increased may also relate to the energetic cost associated with swimming in the resultant higher water velocities^62^.

The variation in activity across diel periods for each species concurs with what is currently known^32,34,65^, however, variation in orientation of activity over diel periods has not been reported previously. For *M. ikei* and *P. novemaculeata*, the variations in orientation of activity may coincide with the movement of small migratory prey. In lentic environments, zooplankton often migrate horizontally on a daily basis, moving up into the shallow littoral zone at night^66^, which can also drive a similar migration in small teleost and invertebrate predators^67^. *Percalates novemaculeata* are active non-benthic predators^68^ and *M. ikei* exhibit both active foraging and opportunistic ambush predatory behaviours^69^. *Percalates novemaculeata* likely hunt up and down the bank in the littoral zone during the night, while, *M. ikei*, which spend the day period along the banks, also move laterally to more prey-dense areas at night. In contrast, *T. tandanus* is a benthic species feeding primarily on benthic macroinvertebrates^70^.

#### Nesting behaviour of *T. tandanus*

*Tandanus tandanus* nests were more abundant below the weir than in the impounded area above the weir. In addition, nests were active for longer below the weir compared with upstream. The implications of this for recruitment success are unclear, however high nest densities and long nest durations implies increased mating opportunities, possibility of multiple broods and possibly longer egg incubation times - all of which may lead to increased numbers and fitness of offspring. Above the weir, the lower number of nests, shorter duration of active nests, asynchronous timing and the avoidance of the deeper areas closer to the weir wall, indicates that the environmental conditions within the weir pool and less favourable for *T. tandanus* nesting.

We found *T. tandanus* nest sites to be distributed non-randomly. *Tandanus tandanus* are known to breed in impoundments and other deep water bodies^36,37^, but our study suggests that they will preferentially breed in riverine conditions when available (i.e. shallow and slow water velocity). The coarser substrate and water velocity found in riverine systems, compared to that of a reservoir, allows greater oxygenation and increase survivorship of demersal fish eggs^71,72^. Among some species there is also a relationship between water temperature, egg size and development^73^. Given the deeper, more lentic environment and cooler water temperatures recorded during nest site surveys, the weir pool effectively reduced preferential nesting area by 400 m of the river.

Above the no-nest area within 200 m upstream of the weir wall, *T. tandanus* had several notable differences in nest site selection compared to downstream conspecifics. Nest sites above the weir showed strongest selection for deeper water (1–2 m) and zero water velocity, compared to strongest selection for shallower water (0–1 m) and slightly higher water velocity (0.01–0.25 m·s^−1^) below the weir. Like many other species of nesting fish^74–76^, it is likely *T. tandanus* select nest sites based on multiple environmental characteristics. Although suitably shallow areas were available above the weir, these areas were likely unsuitable as the substrate was covered in fine silt, or immediately above the weir water velocities were too high. Additionally, areas of low water velocity only occurred in areas of coarse substrate. The reservoir above an instream barrier such as the Nymboida Weir presents a gradient of conditions, gradually returning to riverine conditions from the lentic environment directly above the barrier. This, coupled with the substrate nesting requirements of *T. tandanus*^37,42^, possibly resulted in fish above the weir selecting sub-optimal nest sites. Further research on the relationship between egg development and survivorship and nest site selection is required to complete this assertion.

## Conclusions

We found that small instream barriers, such as low-head weirs, not only act as a physical and ecological barrier to large-scale movement, and therefore potentially breeding migrations, but also to regular small-scale movements and breeding behaviours of non-migratory species. Although the effects of the weir may have been further reaching for *P. novemaculeata* linear movement, the weir only negatively impacted *T. tandanus* nesting within the impounded area. The installation of fish passage infrastructure may reduce the physical barrier to fish, but aside from removal of the weir, no apparent solution for mitigating the ecological effects of the reservoir exists. A solution may become apparent with further research on the interactive effects of environmental variations on fish movement behaviours around instream barriers and on fish reproduction in relation to barriers.

## Supplementary information

Supplementary information.

Supplementary information.

## Data Availability

It is out intention to upload the data used in this study to either the Australian Animal Tracking and Monitoring System (AATAMS) Facility or the Global Biodiversity Information Facility (GFIB). Luke Carpenter-Bundhoo had full access to all the data in the study, and takes responsibility for the integrity of the data and the accuracy of the data analysis.

## References

[1] Grill G (2019). Mapping the world’s free-flowing rivers. Nature.

[2] Poff NL (1997). The natural flow regime. Bioscience.

[3] Yang D (2006). Distribution and movement of Chinese sturgeon, *Acipenser sinensis*, on the spawning ground located below the Gezhouba Dam during spawning seasons. Journal of Applied Ichthyology.

[4] Jackson A, Moser M (2012). Low-elevation dams are impediments to adult Pacific lamprey spawning migration in the Umatilla River, Oregon. North Am. J. Fish Manage..

[5] Crook DA (2015). Human effects on ecological connectivity in aquatic ecosystems: integrating scientific approaches to support management and mitigation. Science of the Total Environment.

[6] de Leaniz CG, Berkhuysen A, Belletti B (2019). Beware small dams, they can do damage, too. Nature.

[7] Tiemann JS, Gillette DP, Wildhaber ML, Edds DR (2004). Effects of lowhead dams on riffle‐dwelling fishes and macroinvertebrates in a midwestern river. Transactions of the American Fisheries Society.

[8] Hänfling B, Weetman D (2006). Concordant genetic estimators of migration reveal anthropogenically-enhanced source-sink population structure in the river sculpin, *Cottus gobio*. Genetics.

[9] Sard NM (2015). Factors influencing spawner success in a spring Chinook salmon (*Oncorhynchus tshawytscha*) reintroduction program. Can J Fish Aquat Sci.

[10] Porto L, McLaughlin R, Noakes D (1999). Low‐head barrier dams restrict the movements of fishes in two Lake Ontario streams. North Am. J. Fish Manage..

[11] Clapp, D. F., Clark, R. D. Jr. & Diana, J. S. Range, activity, and habitat of large, free-ranging brown trout in a Michigan stream. *Transactions of the American Fisheries Society* **119**, 1022-1034, 10.1577/1548-8659(1990)119<1022:RAAHOL>2.3.CO;2 (1990).

[12] Harvey BC (1991). Interactions among stream fishes: predator-induced habitat shifts and larval survival. Oecologia.

[13] Power ME, Matthews WJ, Stewart AJ (1985). Grazing minnows, piscivorous bass, and stream algae: dynamics of a strong interaction. Ecology.

[14] Schaefer JF, Marsh-Matthews E, Spooner DE, Gido KB, Matthews WJ (2003). Effects of barriers and thermal refugia on local movement of the threatened leopard darter, *Percina pantherina*. Environmental Biology of Fishes.

[15] Kaya CM, Kaeding LR, Burkhalter DE (1977). Use of a cold-water refuge by rainbow and brown trout in a geothermally heated stream. The Progressive Fish-Culturist.

[16] Matthews K, Berg N (1997). Rainbow trout responses to water temperature and dissolved oxygen stress in two southern California stream pools. J Fish Biol.

[17] Butler GL, Mackay B, Rowland SJ, Pease BC (2009). Retention of intra-peritoneal transmitters and post-operative recovery of four Australian native fish species. Marine and Freshwater Research.

[18] Koehn JDC (2004). *Cyprinus carpio*) as a powerful invader in Australian waterways. Freshwater biology.

[19] Reinfelds IV, Walsh CT, van der Meulen DE, Growns IO, Gray CA (2013). Magnitude, frequency and duration of instream flows to stimulate and facilitate catadromous fish migrations: Australian bass (*Macquaria novemaculeata* Perciformes, Percichythidae). River Res. Appl..

[20] Pelicice FM, Pompeu PS, Agostinho AA (2015). Large reservoirs as ecological barriers to downstream movements of Neotropical migratory fish. Fish and Fisheries.

[21] Gillette D, Tiemann J, Edds D, Wildhaber M (2006). Habitat use by a Midwestern USA riverine fish assemblage: effects of season, water temperature and river discharge. J Fish Biol.

[22] Persson, L. Temperature-induced shift in foraging ability in two fish species, roach (*Rutilus rutilus*) and perch (*Perca fluviatilis*): implications for coexistence between poikilotherms. *The Journal of Animal Ecology*, 829-839 (1986).

[23] Van Der Kraak, G. & Pankhurst, N. W. *Temperature effects on the reproductive performance of fish*. 159-176 (Cambridge University Press, 1997).

[24] Harris J, Kingsford R, Peirson W, Baumgartner L (2017). Mitigating the effects of barriers to freshwater fish migrations: the Australian experience. Marine and Freshwater Research.

[25] Dudgeon D (2006). Freshwater biodiversity: importance, threats, status and conservation challenges. Biological reviews.

[26] Britto deC, Carvalho S (2013). E. Reproductive migration of fish and movement in a series of reservoirs in the Upper Parana River basin, Brazil. Fisheries Management and Ecology.

[27] Threatened Species Scientific Committee. (ed Department of the Environment.) (Canberra, 2015).

[28] Rowland SJ (1993). *Maccullochella ikei*, an endangered species of freshwater cod (Pisces: Percichthyidae) from the Clarence River system, NSW and *M. peelii mariensis*, a new subspecies from the Mary River system, Qld. Records of the Australian Museum.

[29] Fisheries Scientific Committee. (ed Department of Primary Industries) 1–4 (Port Stephens Fisheries Centre, Nelsons Bay, NSW, 2008).

[30] Harris, J. Impoundment of coastal drainages of southeastern Australia, and a review of its relevance to fish migrations. *Australian Zoologist*, 235–250 (1983).

[31] Stoessel DJ, Morrongiello JR, Raadik TA, Lyon J, Fairbrother P (2018). Is climate change driving recruitment failure in Australian bass, *Macquaria novemaculeata*, in southern latitudes of the species range?. Marine and Freshwater Research.

[32] Koster WM (2015). Movement and habitat use of the freshwater catfish (*Tandanus tandanus*) in a remnant floodplain wetland. Ecology of Freshwater Fish.

[33] Reynolds L (1983). Migration patterns of five fish species in the Murray-Darling River system. Marine and Freshwater Research.

[34] Butler G, Rowland S, Baverstock P, Brook sL (2014). Movement patterns and habitat selection of the Endangered eastern freshwater cod *Maccullochella ikei* in the Mann River, Australia. Endangered Species Research.

[35] Butler GL, Rowland SJ (2009). Using underwater cameras to describe the reproductive behaviour of the endangered eastern freshwater cod *Maccullochella ikei*. Ecology of Freshwater Fish.

[36] Davis, T. L. O. Biology of the freshwater catfish, Tandanus tandanus, Mitchell (pisces: Plotosidae) in the Gwydir River, N.S.W., Australia: with particular reference to the impoundment of this river by the Copeton Dam. PhD thesis, University of New England, (1975).

[37] Lake JS (1967). Rearing experiments with five species of Australian freshwater fishes. I. Inducement to spawning. Marine and Freshwater Research.

[38] Harding DJ (2017). Migration patterns and estuarine aggregations of a catadromous fish, Australian bass (*Percalates novemaculeata*) in a regulated river system. Marine and Freshwater Research.

[39] Espinoza M, Farrugia TJ, Webber DM, Smith F, Lowe CG (2011). Testing a new acoustic telemetry technique to quantify long-term, fine-scale movements of aquatic animals. Fisheries Research.

[40] Jepsen N, Koed A, Thorstad EB, Baras E (2002). Surgical implantation of telemetry transmitters in fish: how much have we learned?. Hydrobiologia.

[41] Wagner GN, Cooke SJ, Brown RS, Deters KA (2011). Surgical implantation techniques for electronic tags in fish. Rev. Fish. Biol. Fish..

[42] Merrick J, Midgley S (1981). Spawning behaviour of the freshwater catfish *Tandanus tandanus* (Plotosidae). Marine and Freshwater Research.

[43] Pusey, B. J., Kennard, M. J. & Arthington, A. H. *Freshwater fishes of North-Eastern Australia*. (CSIRO Publishing, 2004).

[44] Simonson, T. D., Lyons, J. & Kanehl, P. D. Quantifying fish habitat in streams: transects pacing, sample size, and a proposed framework. *North Am. J. Fish Manage*. **14**, 607-615, 10.1577/1548-8675(1994)014<0607:QFHIST>2.3.CO;2 (1994).

[45] Carpenter-Bundhoo, L. *et al*. Reservoir to river: quantifying fine scale fish movements after translocation. *Ecology of Freshwater Fish* **29**, 89–102, 0.1111/eff.12490 (2020).

[46] Campbell HA, Watts ME, Dwyer RG, Franklin CE (2012). V-Track: software for analysing and visualising animal movement from acoustic telemetry detections. Marine and Freshwater Research.

[47] Insol: solar radiation v. 1.2 (R-project, CRAN, 2014).

[48] Bates D, Mächler M, Bolker B, Walker S (2015). Fitting linear mixed-effects models using lme4. Journal of Statistical Software.

[49] Zuur AF, Ieno EN, Elphick CS (2010). A protocol for data exploration to avoid common statistical problems. Methods in Ecology and Evolution.

[50] Furey NB, Dance MA, Rooker JR (2013). Fine-scale movements and habitat use of juvenile southern flounder *Paralichthys lethostigma* in an estuarine seascape. J Fish Biol.

[51] Calenge C (2006). The package “adehabitat” for the R software: A tool for the analysis of space and habitat use by animals. Ecological Modelling.

[52] Conner, L. M. & Plowman, B. W. In *Radio Tracking and Animal Populations* (ed John M. Marzluff) Ch. 10, 275-290 (Academic Press, 2001).

[53] Conner LM, Smith MD, Burger LW (2003). A comparison of distance‐based and classification‐based analyses of habitat use. Ecology.

[54] O’Connor J, Amtstaetter F, Jones M, Mahoney J (2015). Prioritising the rehabilitation of fish passage in a regulated river system based on fish movement. Ecological Management & Restoration.

[55] Januchowski-Hartley SR (2013). Restoring aquatic ecosystem connectivity requires expanding inventories of both dams and road crossings. Frontiers in Ecology and the Environment.

[56] Avery-Gomm S, Rosenfeld JS, Richardson JS, Pearson M (2014). Hydrological drought and the role of refugia in an endangered riffle-dwelling fish, Nooksack dace (*Rhinichthys cataractae* ssp.). Can J Fish Aquat Sci.

[57] Morelli TL (2017). Climate change refugia and habitat connectivity promote species persistence. Climate Change Responses.

[58] Silver P, Cooper JK, Palmer MA, Davis EJ (2000). The arrangement of resources in patchy landscapes: effects on distribution, survival, and resource acquisition of chironomids. Oecologia.

[59] Detar JE, Mattingly HT (2013). Movement patterns of the threatened Blackside Dace, *Chrosomus cumberlandensis*, in two southeastern Kentucky watersheds. Southeast. Nat..

[60] Woolnough DA, Downing JA, Newton TJ (2009). Fish movement and habitat use depends on water body size and shape. Ecology of Freshwater Fish.

[61] Gardner C, Deeming DC, Eady P (2015). Seasonal water level manipulation for flood risk management influences home‐range size of common bream *Abramis brama L*. in a lowland river. River Res. Appl..

[62] Ohlberger J, Staaks G, van Dijk PL, Hölker F (2005). Modelling energetic costs of fish swimming. Journal of Experimental Zoology Part A: Comparative Experimental Biology.

[63] Smith, J. J. & Li, H. W. In Predators and prey in fishes: *Proceedings of the 3rd biennial conference on the ethology and behavioral ecology of fishes, held at Normal*, Illinois, U.S.A., May 19–22, 1981 (eds David L. G. Noakes, David G. Lindquist, Gene S. Helfman, & Jack A. Ward) 173-180 (Springer Netherlands, 1983).

[64] Harris, J. & Rowland, S. *Family Percichthyidae: Australian freshwater cods and basses*. (Reed books, 1996).

[65] Smith JA, Baumgartner LJ, Suthers IM, Taylor MD (2011). Distribution and movement of a stocked freshwater fish: implications of a variable habitat volume for stocking programs. Marine and Freshwater Research.

[66] Burks R, Lodge D, Jeppesen E, Lauridsen T (2002). Diel horizontal migration of zooplankton: costs and benefits of inhabiting the littoral. Freshwater Biology.

[67] Wurtsbaugh W, Li H (1985). Diel migrations of a zooplanktivorous fish (Menidia beryllina) in relation to the distribution of its prey in a large eutrophic lake1. Limnology and Oceanography.

[68] Pusey, B., Kennard, M. J. & Arthington, A. H. *Freshwater fishes of North-eastern Australia*. (CSIRO publishing, 2004).

[69] Butler GL, Wooden IJ (2012). Dietary habits of a large, long-lived endangered Australian percichthyid, the eastern freshwater cod *Maccullochella ikei*. Endangered Species Research.

[70] Davis T (1977). Food habits of the freshwater catfish, *Tandanus tandanus*, Mitchell, in the Gwydir River, Australia, and effects associated with inpoundment of this river by the Copeton Dam. Marine and Freshwater Research.

[71] Kemp P, Sear D, Collins A, Naden P, Jones I (2011). The impacts of fine sediment on riverine fish. Hydrological Processes.

[72] Reiser DW, White RG (1988). Effects of two sediment size-classes on survival of steelhead and chinook salmon eggs. North Am. J. Fish Manage..

[73] Kikko T, Usuki T, Ishizaki D, Kai Y, Fujioka Y (2015). Relationship of egg and hatchling size to incubation temperature in the multiple-spawning fish *Gnathopogon caerulescens* (Honmoroko). Environmental Biology of Fishes.

[74] Bruton M, Boltt R (1975). Aspects of the biology of *Tilapia mossambica* Peters (Pisces: Cichlidae) in a natural freshwater lake (Lake Sibaya, South Africa). J Fish Biol.

[75] Noltie DB, Keenleyside MHA (1987). Breeding ecology, nest characteristics, and nest-site selection of stream- and lake-dwelling rock bass, *Ambloplites rupestris* (Rafinesque). Canadian Journal of Zoology.

[76] Stahr KJ, Kaemingk MA, Willis DW (2013). Factors associated with bluegill nest site selection within a shallow, natural lake. Journal of freshwater ecology.

