# Supplementary material for: Effects of a low-head weir on multi-scaled movement and behavior of three riverine fish species
Source: Sci Rep. 2020 Apr 22;10:6817. doi: 10.1038/s41598-020-63005-8 (PMC7176731; doi:10.1038/s41598-020-63005-8)
Supplement: Supplementary file 1 — Supplementary information. [file 41598_2020_63005_MOESM1_ESM.docx]

Title: Effects of a low-head weir on multi-scaled movement and behavior of three riverine fish species

Authors: Luke Carpenter-Bundhoo, Gavin L. Butler, Nick R. Bond, Stuart E. Bunn, Ivars V. Reinfelds, Mark J. Kennard

**Apendicies**

Appendix 1 – Table of morphology, and tagging and release details of *M. ikei, P. novemaculeata* and *T. tandanus* in the Nymboida River over 2016/2017.

| ID | Species | Sex | Length (mm) | Year released | Release site |
| --- | --- | --- | --- | --- | --- |
| 15775 | *M. ikei* | M | 460 | 2016 | Downstream |
| 15783 | *M. ikei* | F | 461 | 2016 | Downstream |
| 15786 | *M. ikei* | F | 420 | 2016 | Upstream |
| 15986 | *M. ikei* | F | 686 | 2017 | Downstream |
| 30187 | *M. ikei* | M | 363 | 2017 | Upstream |
| 30190 | *M. ikei* | F | 534 | 2017 | Downstream |
| 30195 | *M. ikei* | M | 374 | 2017 | Downstream |
| 30198 | *M. ikei* | M | 380 | 2017 | Upstream |
| 30201 | *M. ikei* | M | 355 | 2017 | Downstream |
| 30202 | *M. ikei* | M | 483 | 2017 | Downstream |
| 30203 | *M. ikei* | F | 403 | 2017 | Upstream |
| 30204 | *M. ikei* | F | 398 | 2017 | Upstream |
| 30205 | *M. ikei* | F | 417 | 2017 | Upstream |
| 15959 | *P. novemaculeata* | NA | 366 | 2016 | Upstream |
| 15965 | *P. novemaculeata* | NA | 385 | 2016 | Upstream |
| 15969 | *P. novemaculeata* | NA | 365 | 2016 | Upstream |
| 15983 | *P. novemaculeata* | F | 394 | 2017 | Downstream |
| 15985 | *P. novemaculeata* | F | 380 | 2017 | Upstream |
| 15987 | *P. novemaculeata* | F | 308 | 2017 | Downstream |
| 15988 | *P. novemaculeata* | F | 373 | 2017 | Upstream |
| 23039 | *P. novemaculeata* | NA | NA | 2017 | Upstream |
| 30191 | *P. novemaculeata* | F | 366 | 2017 | Upstream |
| 30197 | *P. novemaculeata* | F | 354 | 2017 | Upstream |
| 15982 | *T. tandanus* | M | 491 | 2017 | Downstream |
| 30186 | *T. tandanus* | F | 538 | 2017 | Upstream |
| 30188 | *T. tandanus* | F | 544 | 2017 | Downstream |
| 30189 | *T. tandanus* | F | 510 | 2017 | Upstream |
| 30192 | *T. tandanus* | M | 520 | 2017 | Downstream |
| 30193 | *T. tandanus* | F | 391 | 2017 | Downstream |
| 30194 | *T. tandanus* | M | 510 | 2017 | Downstream |
| 30196 | *T. tandanus* | F | 514 | 2017 | Downstream |
| 30199 | *T. tandanus* | M | 511 | 2017 | Upstream |
| 30200 | *T. tandanus* | M | 489 | 2017 | Upstream |
| 57878 | *T. tandanus* | F | 353 | 2017 | Upstream |
