# Supplementary material for: Effects of a low-head weir on multi-scaled movement and behavior of three riverine fish species
Source: Sci Rep. 2020 Apr 22;10:6817. doi: 10.1038/s41598-020-63005-8 (PMC7176731; doi:10.1038/s41598-020-63005-8)
Supplement: Supplementary file 2 — Supplementary information. [file 41598_2020_63005_MOESM2_ESM.docx]

Title: Effects of a low-head weir on multi-scaled movement and behavior of three riverine fish species

Authors: Luke Carpenter-Bundhoo, Gavin L. Butler, Nick R. Bond, Stuart E. Bunn, Ivars V. Reinfelds, Mark J. Kennard

**Supplementary material**


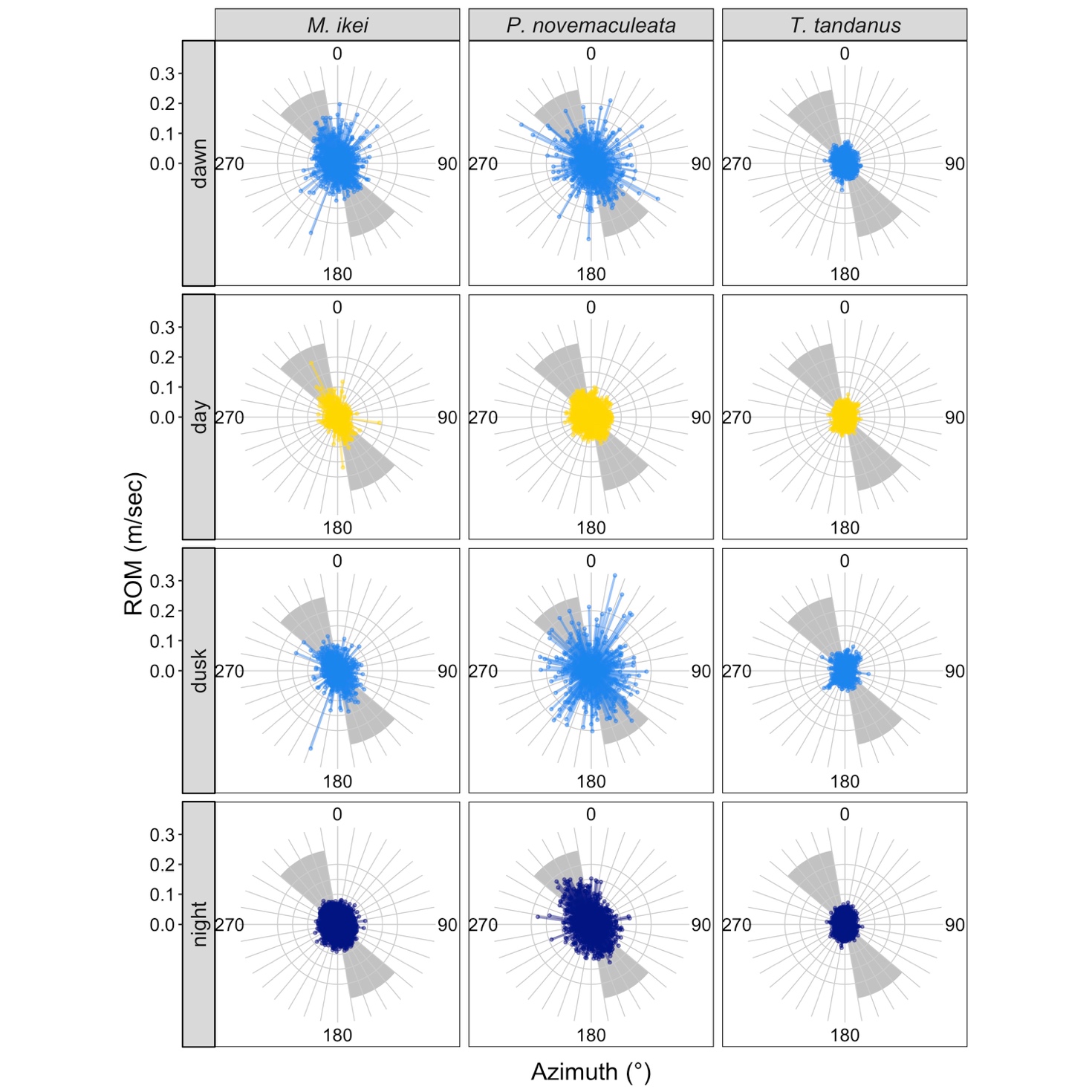


Supplementary material 1. Circular plots of ROM (m.sec^-1^) vs movement directions (azimuth) in each diel period for *M. ikei*, *P. novemaculeata* and *T. tandanus* within the Nymboida Weir pool. Histogram bars are binned at 1 °. Downstream orientation ranges between 310° to 350° and upstream from 130° to 170° (shaded area).
